# Genome Sequence of Bacillus thuringiensis Strain MW, a Freshwater Isolate

**DOI:** 10.1128/MRA.01482-19

**Published:** 2020-01-09

**Authors:** Alexander N. Williams, Kyle S. MacLea

**Affiliations:** a Biotechnology Program, University of New Hampshire, Manchester, New Hampshire, USA; b Biology Program, University of New Hampshire, Manchester, New Hampshire, USA; c Department of Life Sciences, University of New Hampshire, Manchester, New Hampshire, USA; Loyola University Chicago

## Abstract

Bacillus thuringiensis is an agriculturally significant bacterium and common biological pesticide. B. thuringiensis strain MW was isolated from a freshwater stream in Mont Vernon, NH, and sequenced. A draft genome assembly of 5,935,630 bp with a G+C content of 34.86% and an *N*_50_ value of 1,154,949 bp was generated.

## ANNOUNCEMENT

Bacillus thuringiensis is an aerobic, Gram-positive, spore-forming bacterium of the Bacillus cereus group (1). It is well known for its ability to generate δ-endotoxins during sporulation (2, 3), due to which it is commonly used as a biological pesticide targeting insects and other invertebrates (4). Thousands of different B. thuringiensis strains are known, and they produce well over 200 distinct crystal (Cry) toxin proteins. While most commonly found in soil, B. thuringiensis has been isolated from freshwater in Japan (5), the Philippines (6), and Poland (7). Here, we report the genome sequence of a freshwater B. thuringiensis strain (MW) isolated from a stream in the northeastern United States.

B. thuringiensis strain MW was found in a muddy freshwater stream in Mont Vernon, NH, on 3 February 2019 (global positioning system [GPS] coordinates 42.8867870, −71.6385825). Tryptic soy agar (TSA) formulated with 50.0 mg/liter of cycloheximide was used to isolate a white, irregularly shaped colony after incubation at 37°C for 48 h. Genomic DNA was isolated using the QIAamp DNA minikit (Qiagen, Valencia, CA) from 5 ml of inoculated TSA broth grown at 37°C for 24 h. The purified genomic DNA was fragmented and tagged with sequence adapters using the HyperPlus kit (v.3.16, catalog number KR1145; Kapa Biosystems, Wilmington, MA) and sequenced on an Illumina HiSeq 2500 at the Hubbard Center for Genome Studies (Durham, NH). The resulting total of 1,504,486 (250-bp) reads were bioinformatically paired and trimmed using Trimmomatic (v.0.38; settings, paired-end mode with a window size of 4, quality requirement of 15, and minimum read length of 36) (8). A total of 1,275,324 trimmed reads were assembled into a draft genome sequence using SPAdes v.3.13.0 with default settings (9). Contigs of <500 bp were removed, and the remaining contigs were annotated using the NCBI Prokaryotic Genome Annotation Pipeline (PGAP) v.4.8 (10) and QUAST v.5.0.2 (11). Any nonbacterial contaminants identified by PGAP were removed from the final assembly, which had a complete genome size of 5,935,630 bp across 64 contigs, with an estimated sequence coverage of 54×, 4 contigs greater than 1 Mbp (including the largest contig of 1,344,345 bp and the *N*_50_ of 1,154,949 bp), and a G+C content of 34.86%. In total, 6,141 genes, 6,023 coding sequences, 296 pseudogenes, 11 complete rRNAs, 93 tRNAs, and 5 noncoding RNAs were identified by PGAP analysis.

Assignment of strain MW to the species B. thuringiensis was done using BLAST (BLASTn with default settings) (12), average nucleotide identity (99.10% using EzBioCloud) (13), and the NCBI SRA Taxonomy Analysis Tool (STAT; default settings) (available on the NCBI Sequence Read Archive “Analysis” tab). Strain MW was found to be most closely related to B. thuringiensis serovar fukuokanensis (serotype 3a:3d:3e), which has known mosquitocidal δ-endotoxins (14). An initial search for crystalline pesticidal genes in the genome of strain MW found a putative Cry5Ba protein, which has been shown to target hymenopterans, including fire ants, in strain PS86Q3 (15, 16).

### Data availability.

This Bacillus thuringiensis whole-genome shotgun project has been deposited in DDBJ/ENA/GenBank under accession number SUPP00000000. The version described in this paper is the first version, SUPP01000000. The raw Illumina data from BioProject accession number PRJNA534292 were submitted to the NCBI Sequence Read Archive (SRA) under experiment accession number SRX6871116.

## References

[1] Rasko DA, Altherr MR, Han CS, Ravel J 2005 Genomics of the *Bacillus cereus* group of organisms. FEMS Microbiol Rev 29:303–329. doi:10.1016/j.fmrre.2004.12.005. 15808746

[2] Heimpel AM 1967 A critical review of *Bacillus thuringiensis* var. *thuringiensis* Berliner and other crystalliferous bacteria. Annu Rev Entomol 12:287–322. doi:10.1146/annurev.en.12.010167.001443. 5340721

[3] Aronson AI, Beckman W, Dunn P 1986 *Bacillus thuringiensis* and related insect pathogens. Microbiol Rev 50:1–24. 3007957 10.1128/mr.50.1.1-24.1986 PMC373051

[4] Lüthy P, Cordier J, Fischer H 1982 *Bacillus thuringiensis* as a bacterial insecticide: basic considerations and application, p 35–74. *In* Kurstak E (ed), Microbial and viral pesticides. Marcel Dekker, New York, NY.

[5] Ichimatsu T, Mizuki E, Nishimura K, Akao T, Saitoh H, Higuchi K, Ohba M 2000 Occurrence of *Bacillus thuringiensis* in fresh waters of Japan. Curr Microbiol 40:217–220. doi:10.1007/s002849910044. 10688688

[6] Padua LE, Gabriel BP, Aizawa K, Ohba M 1981 *Bacillus thuringiensis* isolated from the Philippines. Philipp Entomol 5:199–208.

[7] Bartoszewicz M, Czyżewska U 2017 Spores and vegetative cells of phenotypically and genetically diverse *Bacillus cereus sensu lato* are common bacteria in fresh water of northeastern Poland. Can J Microbiol 63:939–950. doi:10.1139/cjm-2017-0337. 28930645

[8] Bolger AM, Lohse M, Usadel B 2014 Trimmomatic: a flexible trimmer for Illumina sequence data. Bioinformatics 30:2114–2120. doi:10.1093/bioinformatics/btu170. 24695404 PMC4103590

[9] Bankevich A, Nurk S, Antipov D, Gurevich AA, Dvorkin M, Kulikov AS, Lesin VM, Nikolenko SI, Pham S, Prjibelski AD, Pyshkin AV, Sirotkin AV, Vyahhi N, Tesler G, Alekseyev MA, Pevzner PA 2012 SPAdes: a new genome assembly algorithm and its applications to single-cell sequencing. J Comput Biol 19:455–477. doi:10.1089/cmb.2012.0021. 22506599 PMC3342519

[10] Tatusova T, DiCuccio M, Badretdin A, Chetvernin V, Nawrocki EP, Zaslavsky L, Lomsadze A, Pruitt KD, Borodovsky M, Ostell J 2016 NCBI Prokaryotic Genome Annotation Pipeline. Nucleic Acids Res 44:6614–6624. doi:10.1093/nar/gkw569. 27342282 PMC5001611

[11] Gurevich A, Saveliev V, Vyahhi N, Tesler G 2013 QUAST: quality assessment tool for genome assemblies. Bioinformatics 29:1072–1075. doi:10.1093/bioinformatics/btt086. 23422339 PMC3624806

[12] Altschul SF, Gish W, Miller W, Myers EW, Lipman DJ 1990 Basic Local Alignment Search Tool. J Mol Biol 215:403–410. doi:10.1016/S0022-2836(05)80360-2. 2231712

[13] Yoon S-H, Ha S-M, Kwon S, Lim J, Kim Y, Seo H, Chun J 2017 Introducing EzBioCloud: a taxonomically united database of 16S rRNA gene sequences and whole-genome assemblies. Int J Syst Evol Microbiol 67:1613–1617. doi:10.1099/ijsem.0.001755. 28005526 PMC5563544

[14] Lee HK, Gill SS 1997 Molecular cloning and characterization of a novel mosquitocidal protein gene from *Bacillus thuringiensis* subsp. *fukuokaensis*. Appl Environ Microbiol 63:4664–4670. 9406385 10.1128/aem.63.12.4664-4670.1997 PMC168788

[15] Payne JM, Kennedy MK, Randall JB, Meier H, Uick HJ, Foncerrada L, Schnepf HE, Schwab GE, Fu J January 1997 *Bacillus thuringiensis* toxins active against hymenopteran pests. U.S. patent 5,596,071.

[16] Zeigler DR 1999 Bacillus Genetic Stock Center catalog of strains, seventh ed, part 2: *Bacillus thuringiensis* and *Bacillus cereus*. Bacillus Genetic Stock Center, Columbus, OH.

